# *Sir*-*2.1* mediated attenuation of α-synuclein expression by Alaskan bog blueberry polyphenols in a transgenic model of *Caenorhabditis elegans*

**DOI:** 10.1038/s41598-018-26905-4

**Published:** 2018-07-05

**Authors:** Malabika Maulik, Swarup Mitra, Skyler Hunter, Moriah Hunstiger, S. Ryan Oliver, Abel Bult-Ito, Barbara E. Taylor

**Affiliations:** 1 Department of Chemistry and Biochemistry, University of Alaska Fairbanks, Fairbanks, AK, USA; 2 Department of Biology and Wildlife, University of Alaska Fairbanks, Fairbanks, AK, USA; 3 Department of Pharmacology and Toxicology, The Research Institution on Addictions, The State University of New York at Buffalo, Buffalo, NY, USA; 4 Department of Biological Sciences and College of Natural Sciences and Mathematics, California State University, Long Beach, Long Beach, CA, USA

## Abstract

Misfolding and accumulation of cellular protein aggregates are pathological hallmarks of aging and neurodegeneration. One such protein is α-synuclein, which when misfolded, forms aggregates and disrupts normal cellular functions of the neurons causing Parkinson’s disease. Nutritional interventions abundant in pharmacologically potent polyphenols have demonstrated a therapeutic role for combating protein aggregation associated with neurodegeneration. The current study hypothesized that Alaskan bog blueberry (*Vaccinum uliginosum*), which is high in polyphenolic content, will reduce α-synuclein expression in a model of *Caenorhabditis elegans* (*C*. *elegans*). We observed that blueberry extracts attenuated α-synuclein protein expression, improved healthspan in the form of motility and restored lipid content in the transgenic strain of *C*. *elegans* expressing human α-synuclein. We also found reduced gene expression levels of *sir*-*2.1* (ortholog of mammalian Sirtuin 1) in blueberry treated transgenic animals indicating that the beneficial effects of blueberries could be mediated through partial reduction of sirtuin activity. This therapeutic effect of the blueberries was attributed to its xenohormetic properties. The current results highlight the role of Alaskan blueberries in mediating inhibition of *sir*-*2.1* as a novel therapeutic approach to improving pathologies of protein misfolding diseases. Finally, our study warrants further investigation of the structure, and specificity of such small molecules from indigenous natural compounds and its role as sirtuin regulators.

## Introduction

Neurodegeneration can lead to a multitude of pathological conditions that primarily affects the nervous system^1,2^. The debilitating conditions resulting from progressive degeneration and/or death of neurons can lead to impairments in movement, memory processing, affective behaviors and emotional functioning^3,4^. Several such diseases are classified as protein misfolding diseases in which certain proteins become structurally abnormal and fail to fold into their normal configuration. In the misfolded state, these proteins can become toxic and disrupt normal cellular processes^5–8^. One such protein is α-synuclein whose original function is vesicular transportation in the pre-synaptic terminal of neurons^9,10^. Misfolding of α-synuclein leads to its over aggregation and accumulation^11,12^. This is characterized by formation of small cytoplasmic α -synuclein inclusions in the neurons known as Lewy bodies, which is one of the key pathological hallmarks of Parkinson’s disease^13^. Similar molecular pathologies are also characteristic signatures of disorders like Lewy body dementia and multiple system atrophy^14–16^. Despite significant strides in unraveling some of the cellular mechanisms contributing to these disorders, treatment strategies have been elusive. Development of new therapeutics are, therefore, of utmost public health priority.

There is substantial evidence that specific dietary regimen and plant-based supplementation is closely intertwined with the aging process and related disorders including neurodegeneration^17,18^. Phytochemically, fruits and vegetables are rich sources of polyphenolic compounds like anthocyanins and flavonoids^19,20^. The health promoting effects of the plant-based products could be due to interactions of the active phytochemical components with key cellular machinery involving inflammation, transcription and antioxidant systems^21–23^. Most of these homeostatic cellular mechanisms are disrupted in aging and neurodegeneration^24,25^. At the cellular level, sirtuins, a family of Class III NAD+ dependent histone deacetylases, are implicated in several aging, metabolic and neurodegenerative pathways^26,27^. Though most of its substrates are histones, they also act as upstream regulators for transcription factors such as FOXO and P53^28,29^. Dietary polyphenols such as resveratrol and catechins are known to interact with sirtuins in modulating biochemical and signaling pathways^30,31^. These natural compounds have demonstrated a therapeutic role in various neurodegenerative disorders and improving age-related cognitive decline^17,32^. Blueberries in particular have been shown to improve or reverse age related deficits in both animal and clinical studies^33–39^. The wild Alaskan bog blueberry (*Vaccinum uliginosum*) is a popular subsistence species endemic to the Arctic region. These Alaskan berries contain elevated levels of polyphenolic compounds (phenolic, anthocyanin and flavonoids) compared to commercially grown and temperate counterparts^40,41^. We have previously established the beneficial effects of these endemic berries in improving markers of aging and neuronal health in both *in*-*vitro* and *in*-*vivo* model systems^42,43^. However, there has been no study to address the mechanistic role of berry phytochemicals in eliminating toxic misfolded proteins, a key pathological marker and endpoint for drug targets.

*Caenorhabditis elegans* (*C*. *elegans*) is a very popular genetic model organism due to its ease of use, well defined aging properties, easy visualization and robust genetic malleability^44,45^. Over the last decade, this model has also been explicitly used in studying age- and brain-related disorders, particularly protein misfolding diseases^46,47^. In *C*. *elegans*, *sir*-*2.1* (ortholog of mammalian Sirtuin 1) is a conserved regulator of aging mechanisms^48^. We selected an α-synuclein expression/aggregation strain of *C*. *elegans*, OW13, which allows easy investigation and quantification of protein inclusions *in vivo*^49^. We hypothesized that when treated with Alaskan bog blueberry extracts and polyphenolic fractions, the animals will have reduced protein expression concomitant with improved behavioral motility and lifespan. We also hypothesized that this attenuation of protein expression is due to the interaction of polyphenols with *sir*-*2.1*.

## Materials and Methods

### Strains, maintenance and synchronization

*C*. *elegans* wild-type Bristol N2 and OW13 [unc54P::α-synuclein::YFP + unc-119(+)] strains used for the study were acquired from the Caenorhabditis Genetics Center (University of Minnesota). OW13 animals expresses human α-synuclein in its body wall muscle. The YFP tag is attached to the C-terminal of α-synuclein. The animals were cultured on nematode growth medium (NGM) plates using standard procedures^50^ and maintained at 22 °C. The plates were seeded with *Escherichia coli* (*E. coli*) strain OP-50-1 or HT115 as appropriate for the experiment. The synchronous population was created using the standard egg-lay method (allowing 20–30 animals to lay eggs for 4–6 hrs and then removing the adults at 22 °C) or by hypochlorite treatment (2% sodium hypochlorite and 0.5 M NaOH)^51^. All experimental plates (except those used for fecundity assessment) contained 0.04 μg/ml Fluorodeoxyuridine (FUdR) (Sigma) for progeny control.

### Berry extract preparation

Wild specimens of Alaskan bog blueberries (*Vaccinum uliginosum*) were collected from interior Alaska. Crude extracts were prepared using 80% aqueous acetone and a rotary evaporator^42^. The specific polyphenolic fractions of anthocyanins and proanthocyanidins from the berries were extracted in the laboratory of Dr. Mary Ann Lila at Plants for Human Health Institute, North Carolina and kindly shared by Dr. Thomas Kuhn from the University of Alaska Fairbanks. All extracts were aliquoted and stored at −80 °C until further use. Instead of an extracted fraction of chlorogenic acid from blueberries, a pure form (Sigma) was used for the study to avoid contamination with minor flavonols obtained during the fractionation process^38^. All the specific fractions were reconstituted in deionized water with 0.1% DMSO to achieve a concentration of 400 μg/ml. A fixed volume of deionized water with 0.1% DMSO was used as control. Total phenolic content and total anthocyanin content was measured as per the methods described in^42^ (Supplemental Table 1).

### Treatment administration

To administer the Alaskan berry treatments (both crude and fractions), a fixed volume of desired concentration was prepared in deionized water (with 0.1% DMSO for fractions) and spread on top of NGM plates seeded with live OP50-1/HT115 *E*. *coli* bacteria. The control plates were spread only with a fixed volume of deionized water (with 0.1% DMSO for fraction study). The bacteria were cultured in Miller Luria-Bertani (LB) broth containing 50 μg/ml streptomycin or carbenicillin as appropriate for the experiment. The experimental population was transferred manually with a platinum pick at late larval L4 stage allowing the animals to ingest the extract. Subsequently, the animals were transferred every other day to a fresh plate with the same constituents until the experiments were performed. OW13 animals were treated with 0, 100, 200, or 400 μg/ml of crude berry extract or 0 and 400 μg/ml of the polyphenolic fractions. A single dose of 400 μg/ml of crude extracts/polyphenolic fractions was selected predominantly because the dose-dependent study produced consistent attenuating effects at 400 μg/ml. Treatment started at larval stage L3 (for lipid staining) and L4 stage (for all other experiments) and continued through days 5, 7, and 12 when animals were tested.

### Lifespan analysis

Following egg lay, synchronous population of 22 L4 animals were transferred into two replicates of each treatment (total of 44 animals). Survival was determined daily by visual observation or gentle prodding with a platinum wire under a dissecting microscope. ‘Bagged’ animals and those that crawled off the plates were censored and removed from the experiment (refer to Supplemental Table 3). Out of 44 animals, 8–12 animals were censored from each experiment. Each experiment was repeated at least three times.

### Motility

The motility of aging animals at time point days 5 and 12 were measured based on a class based (A-B-C) system^52^. The animals moving in a normal sinusoidal pattern were classified as A, the ones which required gentle prodding with a platinum wire were classified as B and those which did not respond or moved just head or tail in response to touch were classified as C.

### Fecundity

Fecundity or viable progeny count was performed by plating age-matched adults on fresh treatment plates until the end of their reproductive phase or death. The eggs laid by the adults were allowed to hatch and develop at 22 °C for 48 h. The number of progeny produced by each individual every day was manually counted under a dissecting microscope.

### RNA interference

RNAi plates were prepared using IPTG and 4X concentrated HT115 bacteria for each gene target (*L4440*, *sir*-*2.1 and daf*-*16*) and covered with foil to protect from light. For each replicate of the experiment, control experiments were performed to compare the knockdown of the fluorescence between *GFP* treatment to empty vector (*L4440***)** (Supplemental Fig. 1). All the bacterial clones were sequenced (Supplemental Table 2).

### Lipid staining

Lipid staining was performed using Nile red dye (Sigma). The stock solution was prepared by dissolving 0.5 mg of the dye in 1 ml of acetone, and then mixed with *E*. *coli* OP50-1^53^. L3 larvae of OW13 animals were added to the treatment plates containing 0.04 μg/ml of FuDR and blueberry extracts (0 and 400 μg/ml) and the imaging was performed at day 7 to monitor the lipid content.

### DCF-DA assay

Reactive oxygen species (ROS) were quantified using H2DCF-DA^42^. In a 96-well plate, 50 μl of live age-synchronous, day 7 animals in M9 buffer were added to each well. Just before loading the plate into the microplate reader, 50 μl of 100 mM 2,7-Dichlorofluorescin diacetate (DCF-DA, Sigma) in M9 was added to the wells. Basal and final fluorescence (after 1 h) was recorded at excitation of 485 nm and emission of 520 nm in a Biotek SynergyTM HT Multi-Mode Microplate Reader. Results were also normalized to total protein concentration using the BCA protein assay (Pierce Biotechnology). Each experiment was repeated three times.

### qPCR Analysis

Total RNA was isolated from ~1000 day-7 adult animals using TRI Reagent (Life Technologies). Quantitative PCR (qPCR) experiments were performed with TaQMan gene expression analysis (Life Technologies) according to the protocol provided by the manufacturer to analyze the amount of mRNA of *sir*-*2.1 and* target genes: *daf*-*16* and *cep*-*1*. qPCR data were analyzed as per Applied Biosystem’s software protocol using *cdc*-42 *and pmp*-3 genes as an internal reference for normalization. The primer set (Life Technologies, Grand Island, NY) used for the experiments were: *sir* 2.1 (Ce02459015_g1), *daf*-*16* (Ce02422838_m1), *cep*-*1* (Ce02410616_g1), *cdc*-42 (Ce02435136_g1) and *pmp*-3 (Ce02485188_m1).

### Protein Extraction and Quantification

Day 7 animals were washed 3 times with distilled water, settled with gravity and then homogenized with a sonicator for 30–60 s in 100–200 µL of a lysis buffer (50 mM Tris-HCl pH 7.5, 150 mM NaCl, 1 mM EDTA, 0.2 mM DTT, 1% Triton X-100,v/v; 10% glycerol, v/v) with a proteinase inhibitor (1 mM PMSF). The lysate was centrifuged at 11,000 × g at 4 °C for 10 min. The supernatant was collected, and protein samples were stored at −80 °C. Protein concentration was determined with the BCA assay kit (Pierce Biotechnology).

### Western Blots

For western blots, 60 μg of total protein was mixed with 4X Laemmli loading buffer (VWR Life Sciences), heated at 100 °C for 10 min, then loaded in 12% Tris-Glycine gels (Invitrogen) consisting of a top 4% stacking gel and a bottom 12% resolving gel, and resolved by electrophoresis at 190 V in a running buffer (25 mM Tris base, 190 mM glycine, and 0.1% SDS (w/v)). Proteins were transferred to a Nitrocellulose membrane (Thermo scientific catalogue 88024) at 75 V for 3 hours, and was blocked at 4 °C with 5% Bovine Serum Albumin (BSA) for 2.5 hours. The membrane was incubated in primary antibody (1:1000 dilution, rabbit anti-GFP, Invitrogen Life Technologies) overnight at 4 °C. The membrane was then washed three times with 1× Tris buffered saline with Tween 20 (TBS-T; 0.05 M Tris-HCl, 0.15 M NaCl, 0.1% (v/v) Tween 20, pH 7.5) + 1% BSA and incubated with a secondary antibody solution (1:10,000 dilution, Horse radish peroxidase conjugated goat anti-rabbit; Invitrogen) for 2.5 hours. After three washes with 1× TBS-T, the membrane was developed with SuperSignal™ West Pico PLUS Chemiluminescent Substrate (Thermofisher scientific) and imaged using a ChemiDoc-It imager at constant exposure settings. For probing with Actin as the loading control, the same membrane was incubated in a stripping buffer (0.1% SDS, w/v; 1% Tween 20, v/v; 200 mM glycine, pH 2.2) for 2 × 10 min, washed twice in PBS, then twice in 1× TBS-T + 1% BSA, prior to being blocked in 5% BSA for 2.5 hours. The membrane was treated to the primary antibody (1:1000 dilution, rabbit anti-Actin, Abcam) and the secondary antibody (1: 10,000 dilution, Horse radish peroxidase conjugated goat anti-rabbit, Invitrogen) and developed similar to the anti-GFP detection. For each treatment, protein samples were pooled from three biological replicates. The experiment was repeated twice.

### Microscopy and image quantification

All images were acquired with a Zeiss LSM 510 laser scanning confocal microscope at constant magnification and exposure settings. Images were analyzed with ImageJ (National Institute of Health). Both α-synuclein and lipid content were quantified based on measurement of total fluorescence. A box of fixed height and width was drawn in the head region of the animal. Relative fluorescent intensity were determined by measuring the average integrated density after subtracting the background. Western blot lanes were imaged using ImageJ by measuring the relative band intensity and normalized to endogenous control- actin.

### Statistical analysis

Statistical analysis was performed in SPSS (Version 22) for motility (ordinal logistic model) and lifespan (Kaplan-Meier log rank survival) experiments. All other statistical analysis was performed in Graph Pad Prism (GraphPad Software, Inc). Treatment effect between groups were tested using one-way or two-way analysis of variance (ANOVA) or t-test. Pairwise comparisons for significant differences between treatment groups were made using Tukey’s t-tests.

### Data availability

All data generated or analysed during this study are included in this published article (and its Supplementary Information files) or are available from the corresponding author on reasonable request.

## Results

### Crude extract of Alaskan bog blueberry attenuated expression of human α-synuclein in *C*. *elegans*

**Figure 1:**
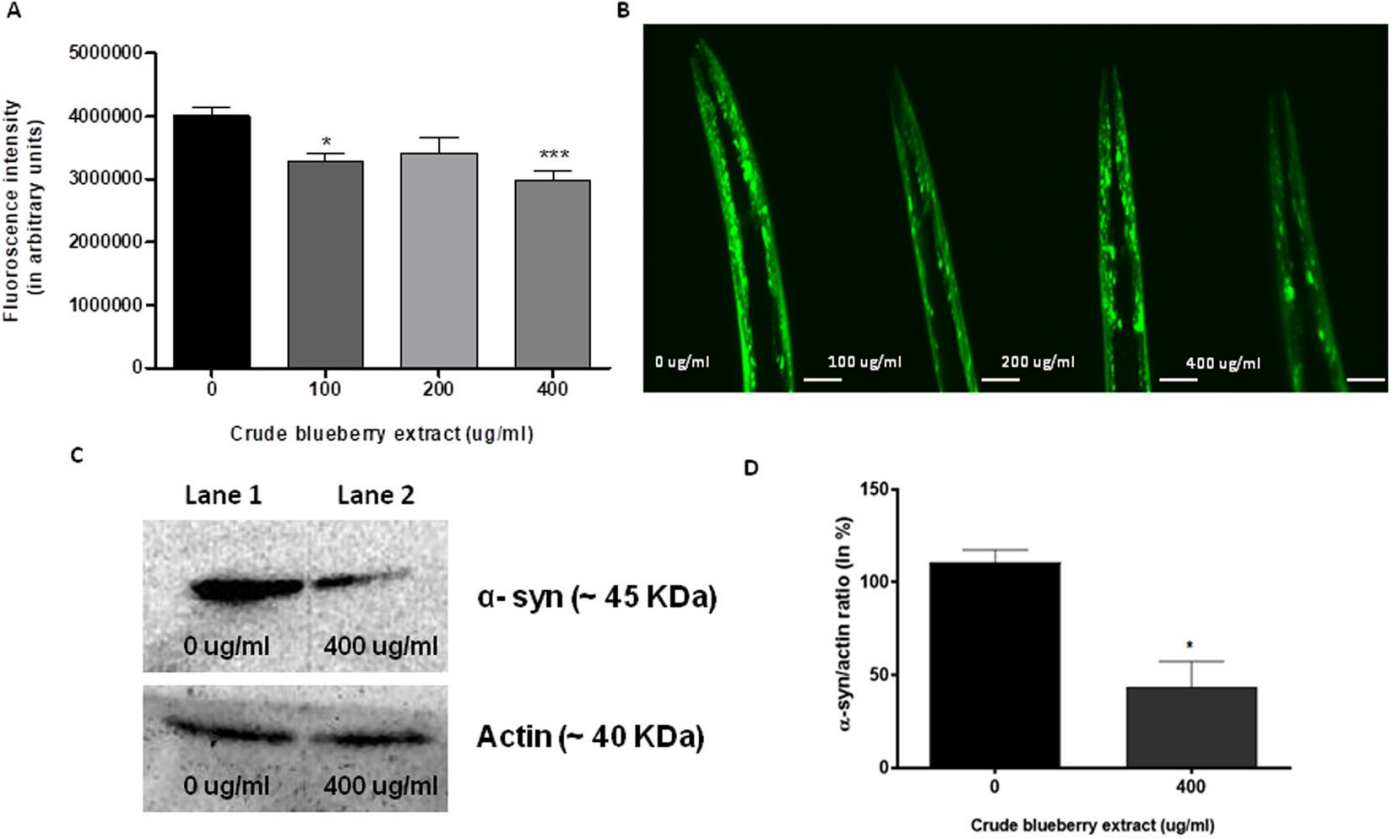
Crude extract of Alaskan blueberry reduced expression of α-synuclein in the OW13 strain of *C*. *elegans*. (**A**) Graphical representation of fluorescence intensity of the OW13 day 7 animals fed on different concentrations of Alaskan blueberry crude extract (0, 100, 200 and 400 μg/ml) from Larval L4 stage. The data represent the mean ± SEM (n = 20–25 animals per group) with significant differences between the control and extract treatments (0 and 100 μg/ml, 0 and 400 μg/ml) *p < 0.01, ***p < 0.001. Dose 200 μg/ml was not significantly different from control (p > 0.4). Each experiment was repeated three times. (**B**) Representative confocal images of the α-synuclein/YFP expression in the head region of day 7 OW13 animals, magnification 40X and scale bar 50 μm. (**C**) Original western blot images of α-synuclein protein of the OW13 day 7 animals fed on concentrations of Alaskan blueberry crude extract (0 and 400 μg/ml). Blots of actin were used as a protein loading control. (**D**) Graphical representation of quantification of α-synuclein protein bands from Western blots. The intensity of α-synuclein protein was normalized to actin and presented as a percentage. Each experiment was repeated twice. For each treatment of each experiment, protein samples were pooled from three biological replicates. The data represent the mean ± SEM with significant differences between control and extract treatments (0 and 400 μg/ml, *p < 0.02).

OW13 animals treated with different doses of crude berry extract (0, 100, 200, 400 μg/ml) were observed on day 7 of the adulthood for protein expression. Two doses of the extract (100 and 400 μg/ml) significantly reduced protein accumulation (One-way ANOVA, p < 0.005). The fluoroscent intensity of the protein accumulation was reduced by about 25–38% (Fig. 1) for the groups administered 100 μg/ml (Tukey’s *post hoc* t-test, p < 0.01) and 400 μg/ml (Tukey’s *post hoc* t-test, p < 0.001) doses. The 200 μg/ml dose was not significantly different from the control (0 μg/ml) (One-way ANOVA, p > 0.4). To further corroborate these findings, western blot analysis was conducted using antibodies probing the YFP part of the α-synuclein::YFP chimeric protein, to measure the protein expression in whole worm extracts on the 7th day of adulthood. The protein expression was reduced by about 40% (Fig. 1) for the 400 μg/ml doses when compared to the control (t-test, p < 0.02).

We also investigated whether these attenuating effects of blueberries were due to secondary responses in *C*. *elegans* resulting from interactions between the live food source, *E*. *coli* OP50-1, and the treatments. Transgenic *C*. *elegans* were grown on Alaskan blueberry treatment with heat-killed OP50-1 *E*. *coli*. In the presence of dead bacteria, blueberry treatment resulted in attenuated protein expression when compared to the untreated control (One-way ANOVA, p < 0.02). A Tukey’s *post hoc* t-test further indicated significant differences between control (0 μg/ml) and treatment groups (100 and 400 μg/ml) (p < 0.01 and p < 0.001, respectively; Supplemental Fig. 2).

### Alaskan bog blueberry polyphenolic fraction reduced expression of human α-synuclein in *C*. *elegans*

**Figure 2:**
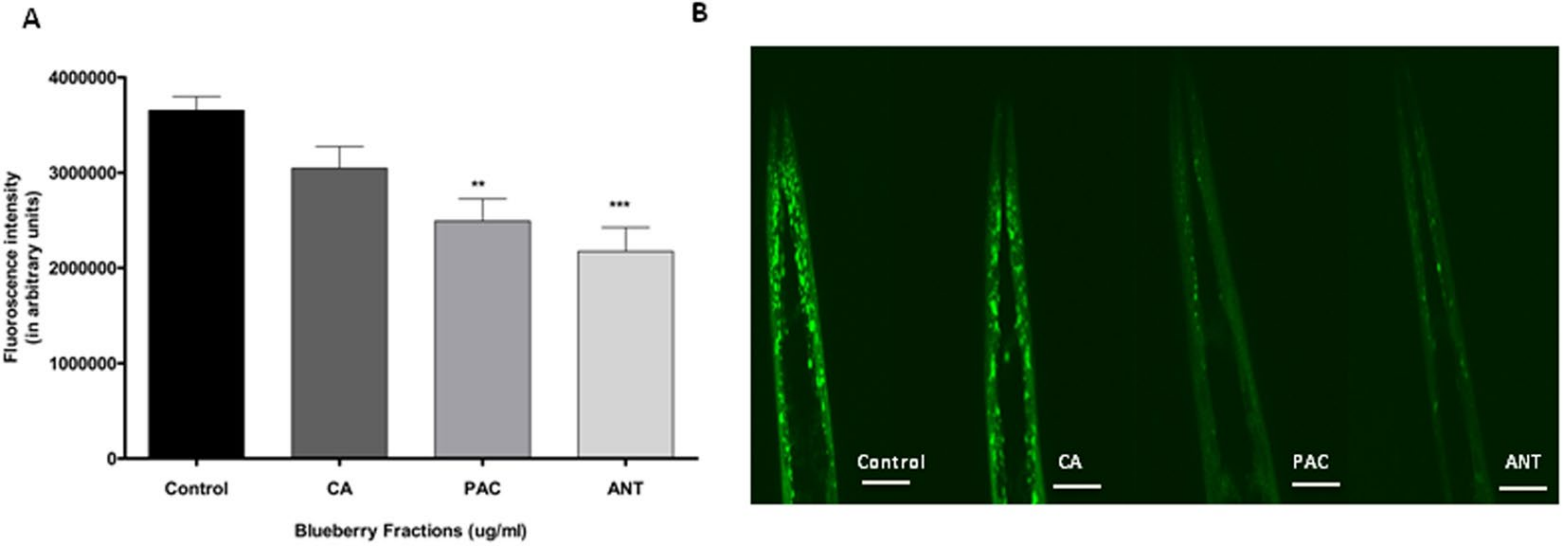
Alaskan blueberry polyphenols reduced expression of α-synuclein in the OW13 strain of *C*. *elegans*. (**A**) Graphical representation of fluorescence intensity of the OW13 day 7 animals fed on different polyphenols found in Alaskan bog blueberry: CA = Chlorogenic acid, PAC = Proanthocyanidins, ANT = Anthocyanins from Larval L4 stage. A concentration of 400 μg/ml was used for all extracts and 0.1% DMSO in deionized water was used as a control. The data represent the mean ± SEM (n = 20–25 animals per group) with significant differences between the control and treatments, **p < 0.001, ***p < 0.0001. Each experiment was repeated three times. (**B**) Representative confocal images of the α-synuclein/YFP expression in the head region of day 7 OW13 animals, magnification 40X and scale bar 50 μm.

Based on our initial findings (Fig. 1), we further aimed to determine which specific fraction reduced the protein expression in *C*. *elegans*. Chlorogenic acid (CA) had no effect on protein expression (One-way ANOVA, p > 0.6) (Fig. 2). However, treatment with both fractions rich in proanthocyanidins (PAC) and anthocyanin (ANT) reduced α-synuclein expression significantly compared to the control (One-way ANOVA, p < 0.0001). The protein accumulation was reduced by about 39% for both PAC (Tukey’s *post hoc* t-test, p < 0.002) and ANT (Tukey’s *post hoc* t-test, p < 0.0002) groups (Fig. 2).

### Alaskan bog blueberry polyphenols reduced human α-synuclein expression in *C*. *elegans* through RNA interference mediating *sir*-*2.1*

**Figure 3:**
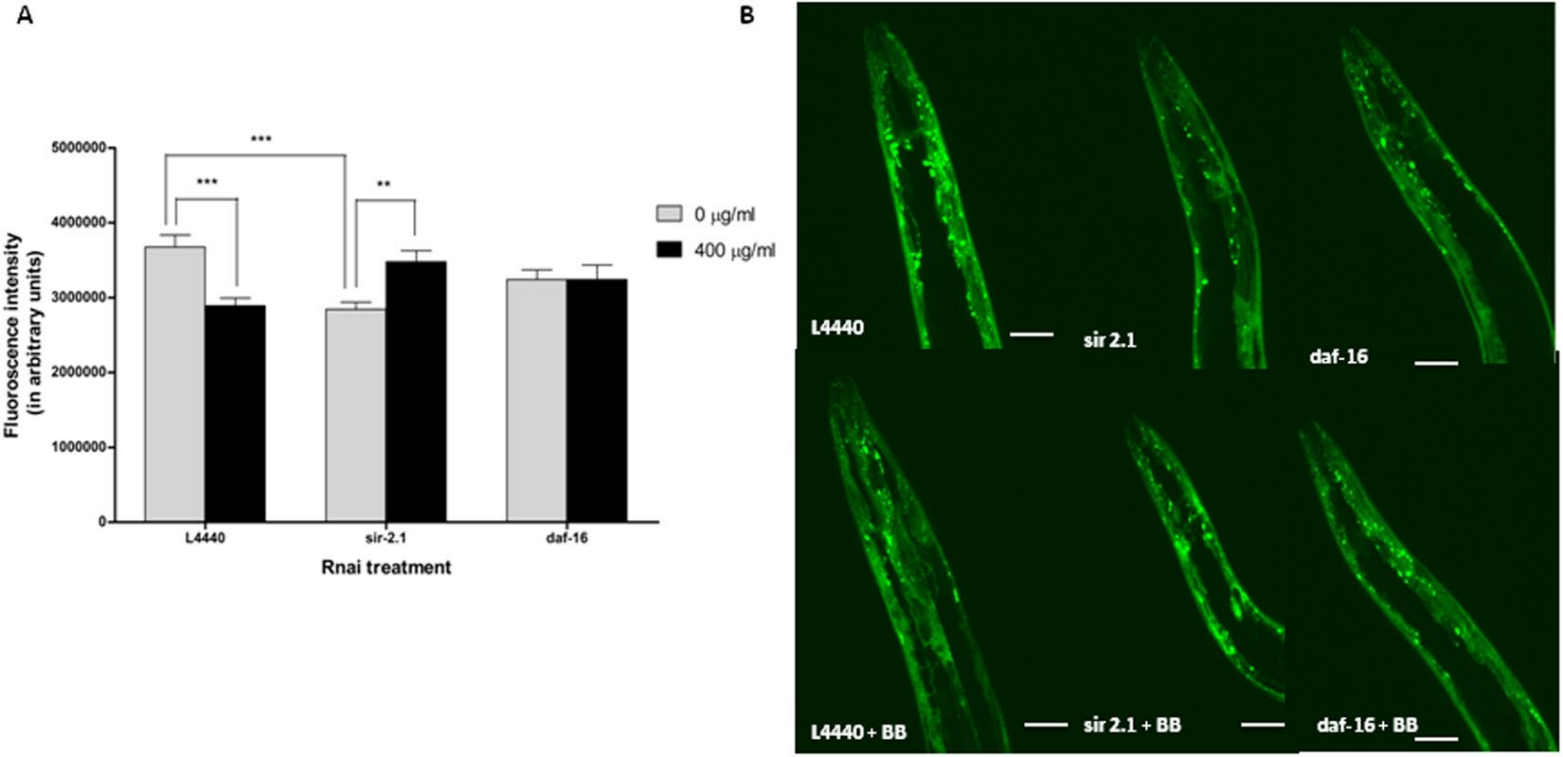
Alaskan blueberry polyphenols reduced expression of α-synuclein in the OW13 strain of *C*. *elegans* through a Sirtuin mediated pathway. (**A**) Graphical representation of fluorescence intensity of the day 7 OW13 animals fed on different genetic RNA interference treatments (*L4440*, *sir*-*2.1 and daf*-*16*) and Alaskan bog blueberry (0 and 400 μg/ml) from larval L4 stage. Empty vector L4440 was considered as a control. The data represent the mean ± SEM (n = 20–25 animals per group) with significant differences between the control and treatments at **p < 0.001 and ***p < 0.0001. Each experiment was repeated three times. (**B**) Representative confocal images of the α-synuclein/YFP expression in the head region of day 7 OW13 animals, magnification 40X and scale bar 50 μm.

No significant effect was observed for RNAi genetic treatment and blueberry treatment (Two-way ANOVA, p > 0.6 and p > 0.7 respectively). However, there was a significant interaction effect between RNAi and blueberry treatment (Two-way ANOVA, p < 0.0003). At day 7 of adulthood, silencing of *sir*-*2.1* decreased protein expression by about 30% (Tukey’s *post hoc* t-test, p < 0.001) (Fig. 3) compared to the empty vector, *L4440*. Also, when treated with blueberry extract (400 μg/ml), the animals fed with *L4440* demonstrated reduced protein expression by 34% (Tukey’s *post hoc* t-test, p < 0.001) (Fig. 3) compared to the animals treated with null control of the extract (0 μg/ml). Most strikingly, when *sir*-*2.1* was silenced in animals and treated with crude blueberry extract (400 μg/ml), the protein expression significantly increased by 29% compared to the control *sir*-*2.1* group (Tukey’s *post hoc* t-test, p < 0.01) (Fig. 3). There was no significance observed within *daf*-*16* groups (0 and 400 μg/ml) (Tukey’s *post hoc* t-test, p > 0.05) as well as between *daf*-*16* and other treatment groups (Tukey’s *post hoc* t-test, p > 0.05).

### Alaskan bog blueberry extract improved motility mediated by *sir*-*2.1* in *C*. *elegans* expressing human α-synuclein

**Figure 4:**
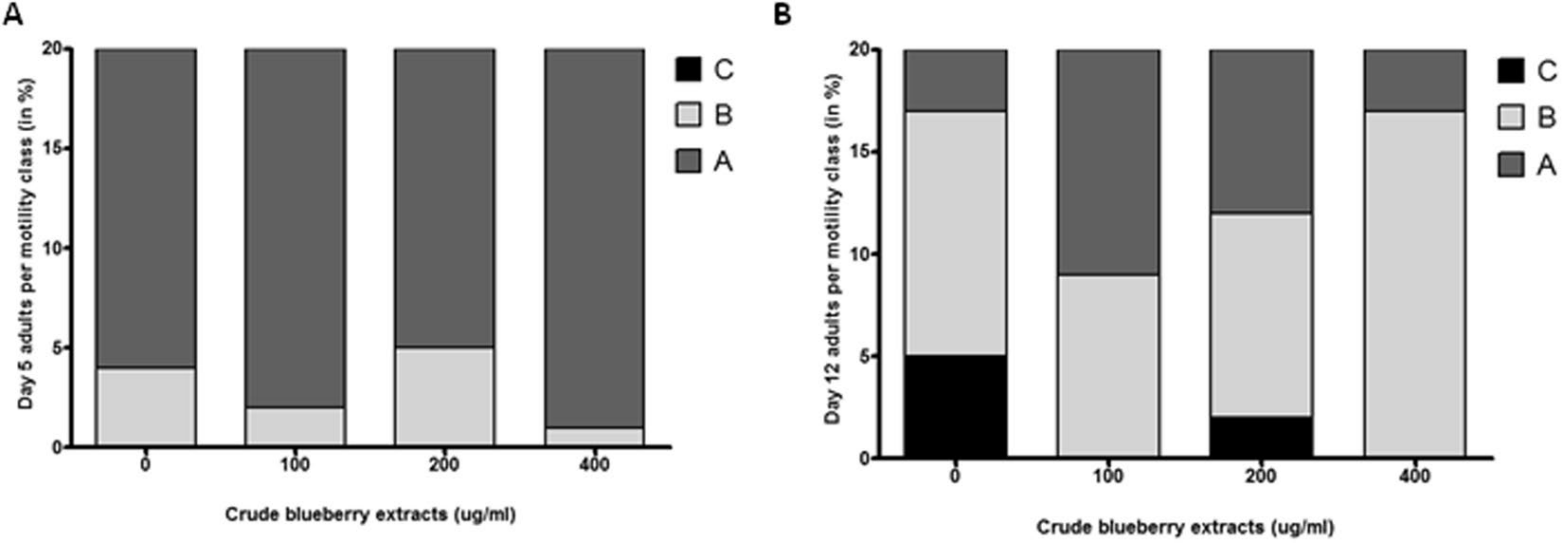
Alaskan bog blueberry polyphenols improved motility in aged (Day 12) OW13 strain of *C*. *elegans*. Percent per motility class of middle age (day 5 adults; (**A**)) and old age (day 12 adults; (**B**)) animals treated with different concentrations of Alaskan bog blueberry extract (0, 100, 200 and 400 μg/ml) from larval L4 stage. Class A animals (dark gray) moved normally and spontaneously, class B animals (light gray) moved abnormally and may have required prodding, and class C animals (black) were unable to move or moved just head or tail in response to touch (n = 20 animals per group). Each experiment was repeated at least three times.

When treated with Alaskan blueberry crude extract (0, 100, 200 and 400 μg/ml), animals at day 5 of adulthood had normal motility compared to the controls (Fig. 4A). However, at day 12 of adulthood, the animals treated with 100 and 400 μg/ml of crude extract of Alaskan blueberry maintained normal motility or had less decline in motility (more Class A and B compared to immobile Class C) compared to the other groups (Ordinal logistic model, p < 0.01) (Fig. 4B).

**Figure 5:**
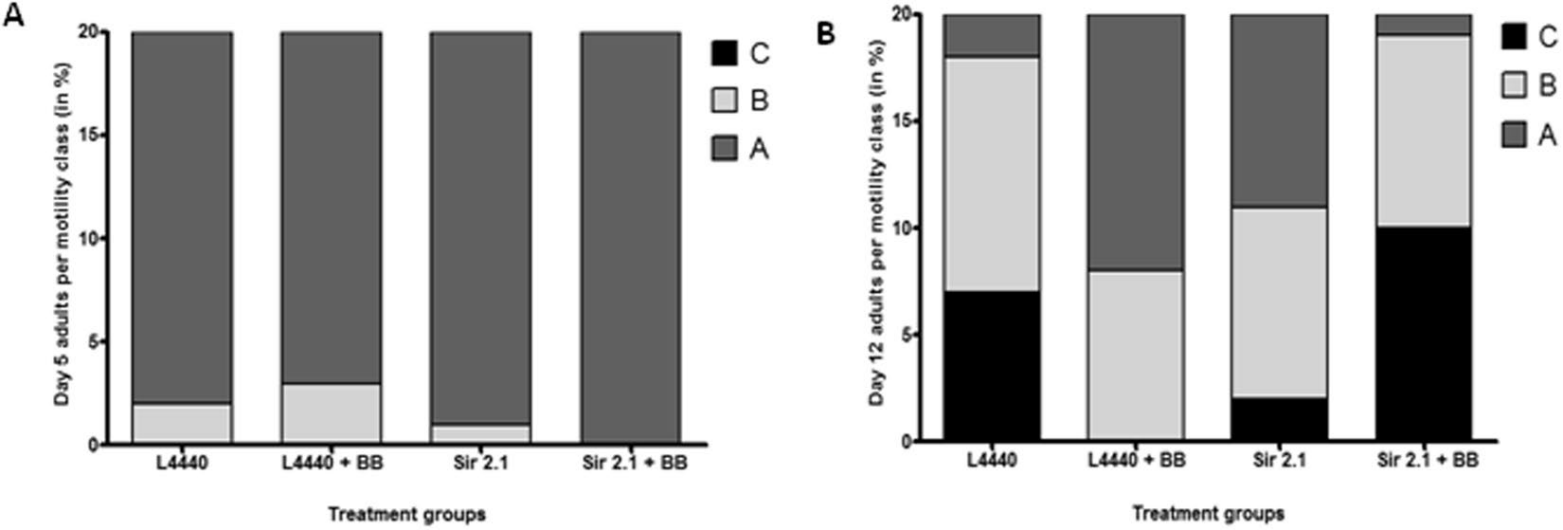
Reduction of *sir*-*2.1* expression reverses the motility improving effects of Alaskan bog blueberry polyphenols in aged (Day 12) OW13 strain of *C*. *elegans*. Percent per motility class of middle age ((day 5 adults; (**A**) and old age (day 12 adults; (**B**)) animals treated with different genetic RNA interference treatments (*L4440*, *sir*-*2.1 and daf*-*16*) and Alaskan bog blueberry extract (0 and 400 μg/ml) from larval L4 stage. Empty vector *L4440* was used as control. Class A animals (dark gray) moved normally and spontaneously, class B animals (light gray) moved abnormally and may have required prodding, and class C animals (black) were unable to move or moved just head or tail in response to touch (n = 20 animals per group). Each experiment was repeated at least three times.

When animals with a silenced *sir*-*2.1* gene were treated with crude extract of Alaskan blueberry, the α-synuclein expression significantly increased. We wanted to further investigate whether silencing *sir*-*2.1* gene had any effect on the animals treated with the Alaskan blueberry extract (0 and 400 μg/ml). Animals in all the groups exhibited normal motility at day 5 (Fig. 5A). However, at day 12 of adulthood the control group treated with blueberries (*L4440* + BB) had more A animals compared to the empty vector control (*L4440*) (ordinal logistic model, p < 0.01) (Fig. 5B). Strikingly, at day 12 of adulthood the group with the *sir*-*2.1* knockdown had lesser class C animals compared to empty vector, *L4440*. Finally, when *sir*-*2.1* knockdown animals were treated with 400 μg/ml of crude extract of Alaskan blueberry, the motility of the animals worsened significantly compared to all other groups (Ordinal logistic model, p < 0.01).

### Alaskan bog blueberry treatments did not affect lifespan or total progeny in *C*. *elegans* expressing human α-synuclein

**Figure 6:**
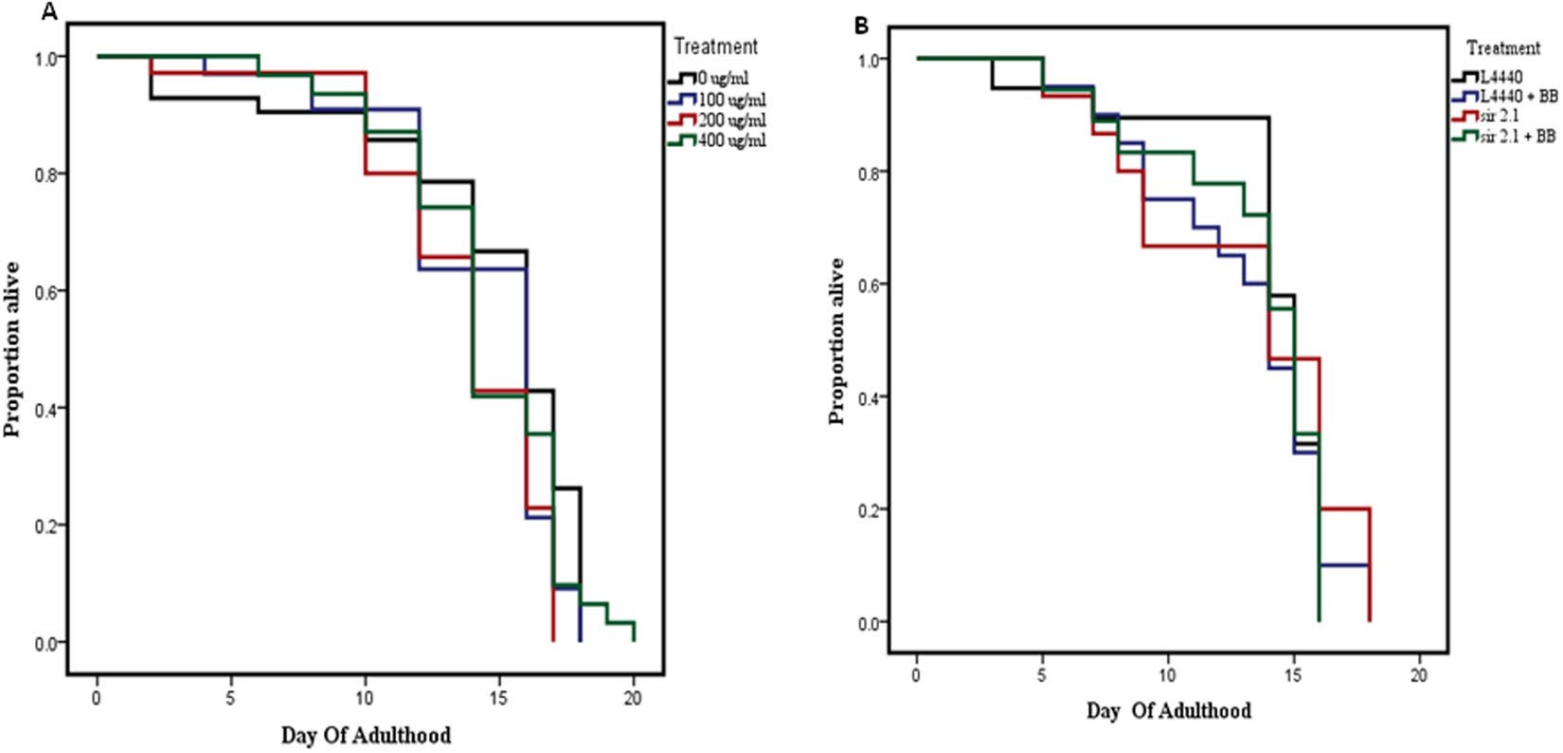
Alaskan bog blueberry did not alter lifespan in OW13 strain of *C*. *elegans*. (**A**) Representative survival curves for animals treated with crude extract of blueberries (0, 100, 200 and 400 μg/ml) from larval L4 stage. There was no significant difference in lifespan extension (p > 0.05; Kaplan–Meier log-rank test) among the groups. Each experiment was repeated at least three times (n = 44 animals per treatment). (**B**) Representative survival curves for animals treated with RNA interference treatments (*L4440 and sir*-*2.1*) and crude extract of blueberries/BB (0 and 400 μg/ml) from larval L4 stage. There was no significant difference in lifespan extension (p > 0.05; Kaplan–Meier log-rank test) among the groups. Each experiment was repeated at least three times (n = 44 animals per treatment).

There was no significant change in mean lifespan among OW13 strain groups treated with tested doses of blueberry extract (0, 100, 200 and 400 μg/ml) (Kaplan Meir statistics, p > 0.05) (Fig. 6A). Interestingly, when animals were treated with genetic treatments (empty vector control: *L4440* and *sir*-*2.1*) and 400 μg/ml of blueberry extract, no significant effect was found in the mean lifespan among the groups (Kaplan Meir statistics, p > 0.05) (Fig. 6B).

**Figure 7:**
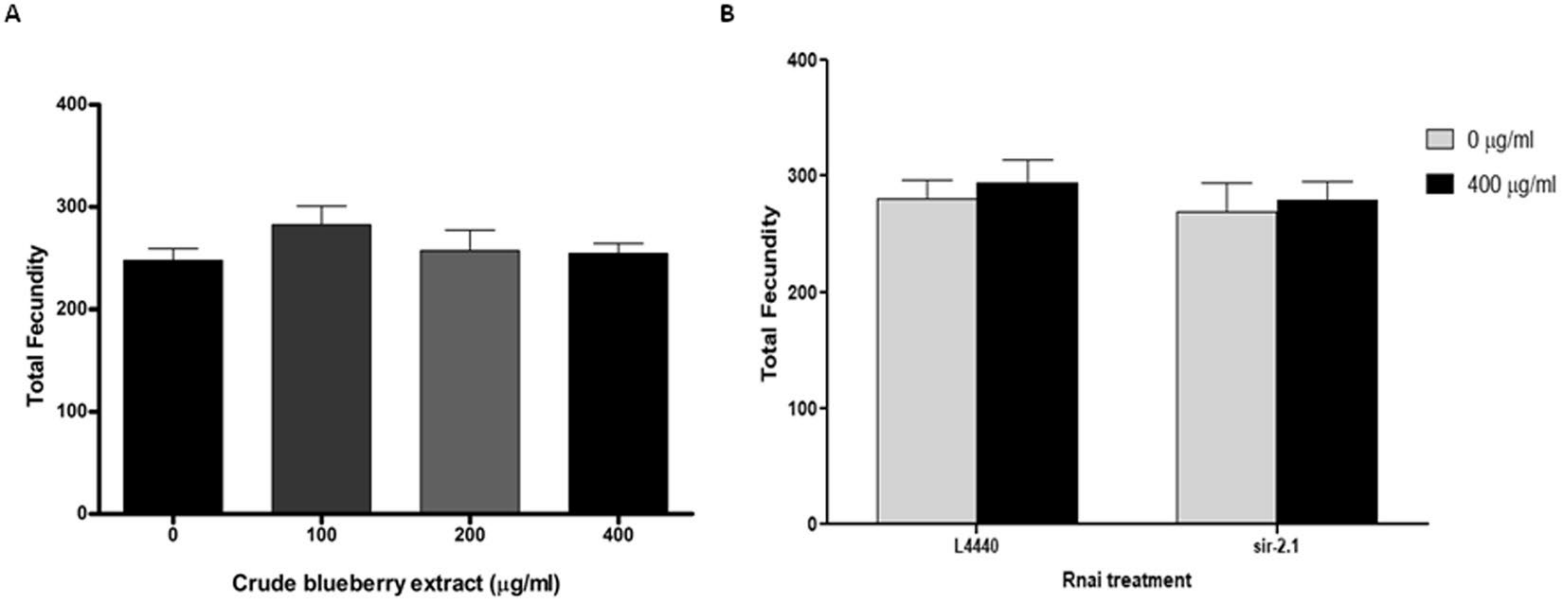
Alaskan bog blueberry and/or RNA interference treatments did not influence total fecundity in OW13 strain of *C*. *elegans*. (**A**) Graphical representation of total fecundity of the OW13 animals fed on different concentrations of Alaskan bog blueberry crude extract (0, 100, 200 and 400 μg/ml) from larval L4 stage. The data represent the mean ± SEM (n = 10 animals per group) with no significant differences among the control and treatments, p > 0.05. Each experiment was repeated three times. (**B**) Graphical representation of total fecundity of the OW13 animals fed on different genetic RNA interference treatments (*L4440 and sir*-*2.1*) and concentrations of Alaskan blueberry crude extract (0 and 400 μg/ml). The data represent the mean ± SEM (n = 10 animals per group) with no significant differences between the control and treatments, p > 0.05. Each experiment was repeated three times.

No significant differences were observed for the total progeny produced between groups treated with Alaskan berry extract (0, 100, 200 and 400 μg/ml) (One-Way ANOVA, p > 0.05) (Fig. 7A). The RNAi treatment groups (*L4440*, *sir*-*2.1*) in combination with crude extract of blueberry (0 and 400 μg/ml) also were not significantly different (Two-way ANOVA, RNAi treatment effect p > 0.05; blueberry treatment effect p > 0.05; interaction effect p > 0.05) (Fig. 7B).

### Alaskan bog blueberry restored lipid content in *C*. *elegans* expressing human α-synuclein

**Figure 8:**
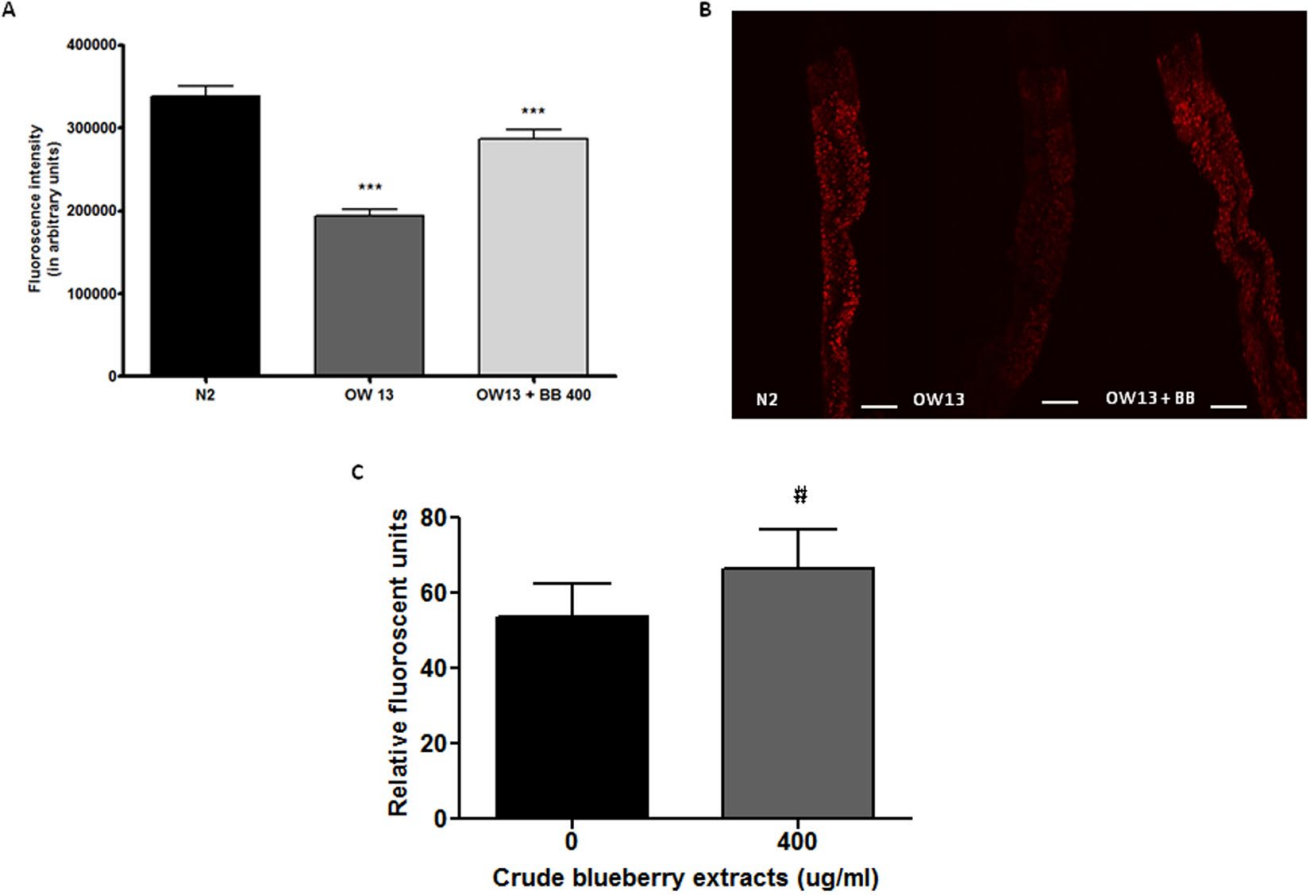
Alaskan bog blueberry increased lipid content by a marginal increase in reactive oxygen species in OW13 strain of *C*. *elegans*. (**A**) Graphical representation for fluorescence intensity for Nile red staining of day 7 wild-type N2 (control) and OW13 animals fed Alaskan bog blueberry extract (0 and 400 μg/ml) from larval L3 stage. The data represent the mean ± SEM (n = 20–25 animals per group) with significant differences between the control and treatments at ***p < 0.001. Each experiment was repeated at least three times. (**B**) Representative confocal images of the intestine of day 7 wild-type N2 (control) and OW13 animals fed Alaskan bog blueberry extract (0 and 400 μg/ml), magnification 20X and scale bar 50 μm. (**C**) The change in endogenous ROS was measured by DCF-DA assay at day 7 of adulthood after treatment with crude extract of blueberries (0 and 400 μg/ml). Bars represent mean ± SEM of each replicate, with three technical replicates for the DCF-DA assay. #p = 0.07.

At day 7 of adulthood, the fat levels of N2 were found to be significantly higher than the OW13 strain, which over expressed α-synuclein in its body (One-way ANOVA, p < 0.001) (Fig. 8A and B). However, treatment with blueberry extract (400 μg/ml) significantly increased total fat content compared to the untreated OW13 group (Tukey’s *post hoc* t-test, p < 0.001) (Fig. 8A and B). At day 7 of adulthood, treatment with 400 μg/ml of blueberry extract, showed a trend towards elevated levels of ROS (t-test, p = 0.07) (Fig. 8C) compared to control.

### Alaskan bog blueberry treatment reduced gene expression levels of *sir*-*2.1* in *C*. *elegans* expressing human α-synuclein

**Figure 9:**
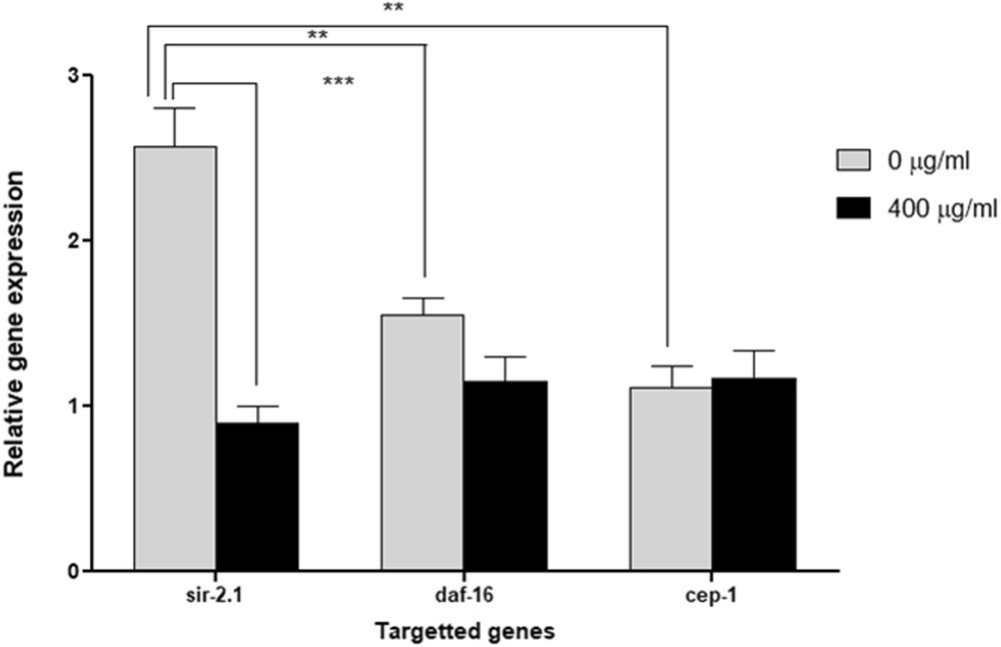
Alaskan bog blueberry extract reduced the gene expression of *sir*-*2.1* in OW13 strain of *C*. *elegans*. qPCR analysis of mRNA levels of *sir*-*2.1*, *daf*-*16 and cep*-*1* (400 μg/ml of crude blueberry or BB extract) in day 7 OW13 animals. qPCR reactions were run in triplicates for each gene. Each experiment was repeated at least three times. The data represent the mean ± SEM at **p < 0.01 and ***P < 0.001.

A significant difference was observed for gene, blueberry treatment and its interaction (Two-way ANOVA, p < 0.03, p < 0.02 and p < 0.002, respectively). At day 7 of adulthood, the gene expression level of *sir*-*2.1* was significantly higher than its downstream targets: *daf*-*16* and *cep*-*1* in untreated control groups (Tukey’s *post hoc* t-test, p < 0.01). When compared to 400 μg/ml blueberry treated group, the relative expression of *sir*-*2.1* was significantly decreased by almost 50% (Tukey’s *post hoc* t- test, p < 0.001) (Fig. 9). However, there were no significant differences (Tukey’s *post hoc* test, p > 0.5) (Fig. 9) between the gene expression levels of *daf*-*16* and *cep*-*1* in animals of both treatment (400 μg/ml) and control (0 μg/ml) groups.

## Discussion

Studies have shown that the effect of natural ‘indigenous diets’ have several health benefits over modern Western diets^54,55^. However, a comprehensive evaluation of the role of photochemical components in influencing cellular mechanisms is sparsely explored in relationship to aging and neurodegeneration. The current study resulted in several novel findings. We report for the first time that crude extract of Alaskan bog blueberry attenuated human α-synuclein aggregation and improved overall motility in a transgenic *C. elegans* model that expressed α-synuclein (OW13). Interestingly, we did not find a dose-dependent effect. Instead, we observed a bimodal or biphasic response in both aggregate reduction and motility enhancement. This pattern of response has also been reported earlier with other phytochemicals in both *in*-*vivo* and *in*-*vitro* models^42,56,57^. The current results are of translational significance since α-synuclein aggregation is a typical pathological feature of protein misfolding diseases such as Parkinson’s disease^4^. A study in a yeast model suggests the therapeutic role of polyphenols like flavonoids in reducing α-synuclein toxicity^58^. Other studies have also revealed the role of flavanoid sub-classes like anthocyanin and proanthocyanidin in conferring protection against oxidative stress *in*-*vitro* and extending lifespan in *C*. *elegans*^38,59,60^. In this current study, anthocyanin and proanthocyanidin fraction of Alaskan blueberry reduced protein over expression in a transgenic strain of *C*. *elegans*. Our results are possibly due to Alaskan blueberries growing under more extreme environmental conditions, such as extreme Arctic temperatures and 24-hours sunlight exposure (UV exposure) during summer months, and thereby having different anthocyanin content^61^. In addition, doses of Alaskan blueberry extract did not influence lifespan and total fecundity in the nematode strain. Studies have shown that improved lifespan can be uncoupled from increased healthspan^62^. In our study, blueberry bimodally improved one measure of healthspan (motility) with no overall effect on the other (fecundity). Importantly, blueberry treatment did not exhibit a uniformly toxic effect because it did not reduce lifespan and did not decrease fertility in this transgenic model of *C*. *elegans*.

In the past decade, sirtuins have received extensive scrutiny in relation to various age-related molecular mechanisms and have been studied across various model systems^63,64^. Particularly, mammalian Sirtuin 1, due to its high abundance in the brain, has drawn much interest in the field of neuroscience including synaptic plasticity and cognition^65,66^. At the same time, research that elucidates the role of natural polyphenols in activating Sirtuin 1 and affecting pathologies of protein misfolding diseases has increased^67–69^. Results from these studies indicate that triggering Sirtuin 1 enhances its deacetylase activity further inducing different pro-survival pathways^70–72^. While the beneficial effect of Sirtuin 1 activation has been comprehensively demonstrated, there are also studies that illuminate the role of Sirtuin 1 inhibition in neuroprotection and improving cellular functions in diseased conditions^73–78^. Genetic and pharmacological studies have found that reduced Sirtuin 1 activity can be beneficial in improving neurodegenerative pathologies in *Drosophila melanogaster*, mammalian cells and mouse models of Huntington’s and Alzheimer’s disease^75,79–82^. In alignment with these studies, we are reporting for the first time the protective effects of Alaskan blueberry through reduction of *sir*-*2.1* gene expression in a model of *C*. *elegans* displaying Parkinson-like molecular pathology. We found that genetically silencing (not deleting) *sir*-*2.1* greatly reduced the α-synuclein expression in OW13. This is further corroborated by the gene expression results suggesting higher gene expression levels of *sir*-*2.1* in the control group compared to the blueberry treated group and other downstream targets in OW13. Higher activity of Sirtuin 1 in metabolically compromised conditions has been linked to higher energy expenditure, which may potentially diminish the effect of the downstream substrates^76,77,83^. In our experimental model of protein overload, lower gene expression levels of downstream substrates such as *daf*-*16* and *cep*-*1* compared to *sir*-*2.1* in untreated controls probably could be justified due to the same mechanism. Strikingly, the basal level of *sir*-*2.1* was also elevated in OW13 when compared to healthy wild-type N2 (Supplemental Fig. 3). The profitable role of Sirtuin 1 activation can depend on physiological state of cells and actually can be detrimental when cells are in stress^76,77^. In our experiments, we observed elevated gene expression levels of *sir*-*2.1* in a model subjected to proteotoxic stress in the form of α-synuclein aggregation.

Research showed how studying alterations in downstream sirtuin substrates, like FOXO, P53 and PGC-1α, can be used as a treatment strategy in protein aggregating diseases^27^. Based on this, we investigated the effects of blueberry extract on two molecular targets of *sir*-*2.1: daf*-*16* (*C*. *elegans* ortholog for mammalian FOXO) and *cep*-*1* (*C*. *elegans* ortholog for mammalian p53). Silencing of *sir*-*2.1* gene in blueberry-treated animals showed an increase in α-synuclein expression. Results also demonstrated very low mRNA levels of *sir-2*.1 in blueberry-treated group compared to controls. However, we also observed that genetic silencing of *daf*-*16* in both control and treatment groups had no effect on the protein expression. Overall, this may indicate that reduced gene expression of *sir*-*2.1* (not complete deletion) could be a potential mechanism through which blueberry exerts its beneficial effect in reducing α-synuclein expression and such pathways may not involve changes in downstream protein targets. Reduction in Sirtuin 1 gene expression through genetic or pharmacological manipulation has been shown to improve cellular symptoms and disease-specific phenotypes in models expressing mutant hungtingtin protein^82^. Smith *et al*. (2014) showed that reducing levels of Sirtuin 1 can result in increased activity of other histone-acetylating enzymes, which can ameliorate the pathology^82^. On the other hand, increased expression of Sirtuin 1 may deacetylate and repress functions of the CREB-binding protein (CBP)^84^ further reducing cell activity. Impaired CREB signalling and reduced transcription also have been reported in cells that overexpress α-synuclein^85^. However, no direct correlations have been drawn on how reduced activity of Sirtuin 1 using pharmacological interventions can possibly improve pathology of synucleinopathies through modulating transcription factors such as CREB. Though we do not speculate on the exact molecular interaction or cross talks, this study strongly advocates for future investigations of such novel mechanisms.

Protein misfolding diseases like Parkinson’s are often associated with alterations in total lipid content^53,86,87^. Increased lipid content has been used as a surrogate measure of improved cellular pathology in studies evaluating natural compounds as potential therapeutics to treat Parkinson’s disease^53,88–90^. However, these studies have not highlighted the exact mechanism behind the effect. We found higher fat levels in the wild-type N2 positive control compared to OW13 strain. The lower fat content in OW13 strain was possibly due to the metabolic burden of protein aggregation. The lipid levels were restored in OW13 animals treated with 400 μg/ml of blueberry extract. This pattern of results is consistent with those shown by others^53,88–90^. However, much to our surprise the fat content and ROS measures did not correlate with each other as anticipated. We found that the blueberry extract marginally increased the ROS levels, a phenomenon previously reported with Alaskan bog blueberries^42^. Xenohormesis is a phenomenon that explains how environmentally stressed plants produce bioactive compounds that confer stress resistance and survival benefits to animals that consume them^91^. Plants, such as blueberries, when subjected to harsh environmental stressors produce secondary metabolites such as polyphenols^41^. Consumption of these plant metabolites by animals can activate stress response pathways other than regular antioxidant effects, thereby improving overall aging and health^92,93^. Additionally, Alaskan blueberries have been shown to inhibit inflammation in neuronal cell cultures through differential biological mechanisms other than their popular image of antioxidant ROS scavengers^43^.

Several studies demonstrate that low levels of ROS can be beneficial in activating signaling pathways to initiate biological processes^94,95^. In *C*. *elegans*, ROS in low concentrations can act as a secondary messenger molecule leading to fat accumulation^96^. This increase in fat content has been found to have contrasting effects on longevity^96^ possibly due to differences in structure of the fat, its localization, or the time point of its accumulation. At day 7 of adulthood, we observed that lipid content was restored in parallel with reduction in α-synuclein expression. Important questions arise from this: What is the mechanistic approach resulting in such ‘beneficial’ increase in lipid levels?, and; are such pathways evolutionarily conserved? Future studies aiming at further characterization of the increased fat content could be useful in answering such questions. Finally, inhibition of Sirtuin 1 has also been found to increase production of ROS due to its positive regulation of antioxidant enzymes^97^. In the current context of investigation, we believe a marginal increase of ROS due to *sir*-*2.1* inhibition by blueberries might activate crucial cell survival pathways overcoming the protein overload. However, such complex interactions between proteostatis, sirtuins, ROS and lipid content should be subjected to further investigation in a variety of cellular and animal models.

## Conclusion

In summary, our results identify a *sir*-*2.1*-mediated mechanism through which Alaskan blueberry polyphenols reduce α-synuclein expression in *C*. *elegans*, while also increasing lipid content and motility. These results indicate that, Sirtuin 1 can act as a double-edge sword in cellular overload conditions due to protein aggregation and partial down regulation of Sirtuin 1 that can restore homeostatic balance. Through the current study, we have also shown how xenohormetic properties of blueberries can trigger the beneficial effect of endogenous ROS signalling molecules. Overall, this study supports the future exploration of photochemical-induced alternative pathways for cell survival in various neurodegenerative disorders.

## Electronic supplementary material

Supplemental figures and tables
