# Supplementary material for: Sir-2.1 mediated attenuation of α-synuclein expression by Alaskan bog blueberry polyphenols in a transgenic model of Caenorhabditis elegans
Source: Sci Rep. 2018 Jul 5;8:10216. doi: 10.1038/s41598-018-26905-4 (PMC6033853; doi:10.1038/s41598-018-26905-4)
Supplement: Supplementary file 1 — Supplemental figures and tables [file 41598_2018_26905_MOESM1_ESM.docx]

**Supplemental figures**

***Sir-2.1* mediated attenuation of α-synuclein expression by Alaskan bog blueberry polyphenols in a transgenic model of *Caenorhabditis elegans***

Malabika Maulik^1^*, Swarup Mitra^1,3^, Skyler Hunter^2^, Moriah Hunstiger^1^, S. Ryan Oliver^1^, Abel Bult-Ito^2^, Barbara E. Taylor^4^

^1^Department of Chemistry and Biochemistry, ^2^Department of Biology and Wildlife,

University of Alaska Fairbanks, ^3^Munroe Meyer Institute, University of Nebraska Medical Center, ^4^Department of Biological Sciences, California State University, Long Beach.

**Figure 1.** Graphical representation of fluorescence intensity of the OW13 animals fed on different RNA interference treatments: *L4440* and *GFP*. The data represent the mean ± SEM (n = 15-20 animals per group) with a significant difference between the control and *GFP* treatment, p<0.01. This was used as a validation plate control for RNA interference experiments.

**Figure 2.** Graphical representation of fluorescence intensity of the OW13 animals fed on different concentrations of Alaskan bog blueberry crude extract (0, 100, 200 and 400 μg/ml) and heat-killed OP-50-1 as a food source. The data represent the mean ± SEM (n = 15-16 animals per group) with significant differences between the control and treatments at *p<0.01 and **p<0.001. Each experiment was repeated two times.

**Figure 3.** qPCR analysis of mRNA levels of *sir-2.1* between wild-type N2 and α-synuclein overexpressing OW13 strain. qPCR reactions were run in triplicates for each strain. Each experiment was repeated at least two times. The data represent the mean ± SEM; *p<0.05.

**Table 1.** Total phenolic content (quantified by Folin–Ciocalteu assay with gallic acid equivalent), and anthocyanin content (quantified by pH-differential with cyanidin-3-glucoside) of Alaskan bog blueberry.

|  | **Value*** |
| --- | --- |
| Total Phenolic content (mg GAE g^-1^ FW) | 198.2±1.4 |
| Total anthocyanin (mg C3G L^-1^ FW) | 285.7±8.2 |

*Data is represented as mean ± SEM for each replicate. Each assay was run in triplicate. The assays were performed following the methods of Scerbak et al., 2016. The values were used for the biochemical quantification of the berry extracts to compare the content reported in Scerbak et al. (2016). The values reported are similar to that reported in previous study.

**Table 2.** Sequencing results of the RNA interference clones. This was performed to ensure the validity of the dsRNA and the sequences were verified using BLAST nucleotide search tool.

| **Name of Clones** | **Sequences** |
| --- | --- |
| ***L4440*** | **GGGGNCANNNNCNATACGGGNCGANTGGNTGACCGGNCCCCCCCCNAAGG**  **CNNGGGNTNANTAACNTTGATATCCCCCTTTAAAAAGAATGAGCAGNTTT**  **TGATGCCAAAAGCTCGATANATCCCGGGAAATAACGCTTCTTACATCGAT**  **GAACGAAATCNNNNACCAAAAATTTCAANNCTCCAAGCGAACGCGCTCAA**  **CACAACTCANAAACATCTAAAGCGGAATTNNNTTATNANNACAATTCNTC**  **ATAACCTTCGCAATGTCTCACCNNNNAANCATCNCACACNTAGTTCCAAA**  **CTTNNCCATCNNATCANCATTCATTCCCACAAANCAGACTTCGANGACCA**  **CAAATCCGNNNNAAAANNNNTTACGTGGANNGCTGGNCCGANNGGNNNNN**  **ANNNNGNATATTCTANNGNNTNATATTCCNNNNTNNCAACAATACNNACT**  **NNNAGATNNTTNATTNNCCNNGAGCCTCNNNCANNNGNNGANNAACNATC**  **CANNNTTCATAAAA** |
| ***sir-2.1*** | **NNNNNNNNNNNNNNNGGCNNNNNNNNNNNNGGGNNNNNNNNNNNNGNNNN**  **NNGNNNNNANNNGCNNNNNNNGNNCNGCANCNNNNNNNNNNNNANNNNNN**  **NNNNNNNNNNNNNNANAGNANNNNANGNNNNNNNNNNNAANNNNNNNNNA**  **ANNNNNTCNGNNNNNACAAAGTCANNTAGCGNNNNNNNNNTNNNNNNNNN**  **NNNNGNNNNNGNNNNGGCNNNNGNNGNNNNATANNNNNNACACNAGCNNN**  **CNCANNNNNNCTCGTGATGAANNNNNCGAGCTANTNNCNACGTTTCTAGA**  **NNNAATGAGCANNCNNTCCCAGGAANGNNNNTTTGANGANNNNNNNNCTG**  **AACAAAAAATNATTANTTGTAAGGCNGNNNNGTAGGTTGTTTAGAAATAT**  **TTTCTAACTTTTGACTTTTTAATTTCCAAAACTACTCACCGCAACACACT**  **TATTGTCATGGAGCATCTCAGTAATGCTCAGCAGCCTTGGATGCTTTATT**  **TTCTTTAGGATAGCGTCCGAATTCCGTGCATCATCGTCATCTTCTGACTT**  **ATGCTTTTGTATTTCCTGGAAATCATTCTTCTCGGAATCCATCAAATCGT**  **CAACGACCGACATTCTCGGTTTCTTCAATGTTGGCTCATCACTCGAATCA**  **TCATTACTCTTCTCTTTCATACATATATTCAAAAAGTTTTCCTGTGAAAT**  **AAGCTGTCGTTTGTTTTGTGAAGGCTTTTGTGATTTCGATTTTCCTTGTT**  **GTTCCATAATTGAATCATAAGATGTAATCAGTTCAGTGAAGGAGCCTCCN**  **AGACTGAAACAGATATCTCTGATGGTGCCATCACAATTTCCGAGCAATTC**  **NATATCCGCATTATNATGTTGGAGGGANTCTCTGTTGANTAGAATTCGTG**  **GACCATTCTTATCNACACNATGAGGAATTNATGCNACTGGTGGAACTTCT**  **ANAGAANATCCNATCACTACNTAANATCTACTTNNTGCTTNNCTTCTGTA**  **NCATGNTGATGAANTNNCNTNCNANANCNNNCNAAGATANANATTNNNTT**  **GATGACNNNNNANNNTTNCATGNNANNCNCNNNTCANACTNNNCNCNANN**  **NNNTNCNNNANTNNNATNTNNCNNNNNCNNNNNNGNNNCNNNTNNNTNNA**  **NANNNGNNNNTNCNNNNNNNNNNANNNCNNNNNNNNNNNNNNNNNNNNNA**  **NNNNNNNNNNNNNNNNNNNNNNNNNNNNNNNNNNNNNNNNNNNNNNNAAN**  **NNNNNNNNNNNNNNNNNNNNNN** |
| ***daf-16*** | **NNNNNNNNNNNNNNNNGNNCNNNNNNNNNNNNGGNCCNNCCTCGAGGTCG**  **ACGGTATCGATAAGCTTGATATCACAAGTTTGTACAAAAAAGCAGGCTTG**  **ATGGAGATGCTGGTAGATCAGGGAACTGATGCATCGTCATCCGCCTCCAC**  **GTCCACCTCATCTGTTTCGAGATTCGGAGCGGACACGTTCATGAATACAC**  **CGGATGATGTGATGATGAATGATGATATGGAACCGATTCCTCGTGATCGG**  **TGCAATACGTGGCCAATGCGTAGGCCGCAACTCGAACCACCACTCAACTC**  **GAGTCCCATTATTCATGAACAAATTCCTGAAGAAGATGCTGACCTATACG**  **GGAGCAATGAGCAATGTGGACAGCTCGGCGGAGCATCTTCAAACGGGTCG**  **ACAGCAATGCTTCATACTCCAGATGGAAGCAATTCTCATCAGACATCGTT**  **TCCTTCGGAAATGTCCGAATCGCCAGACGATACCGTATCGGGAAAAAAGA**  **CAACGACCAGACGGAACGCTTGGGGAAATATGTCATATGCTGAACTTATC**  **ACTACAGCCATTATGGCTAGTCCAGAGAAACGGTTAACTCTTGCACAAGT**  **TTACGAATGGATGGTCCAGAATGTTCCATACTTCAGGGATAAGGGAGATT**  **CGAACAGTTCAGCTGGATGGAAGAACTCGATCCGTCACAATCTGTCTCTT**  **CATTCTCGTTTCATGCGAATTCAGAATGAAGGAGCCGGAAAGAGCTCGTG**  **GTGGGTTATTAATCCAGATGCAAAGCCAGGAAGGAATCCACGGCGTACAC**  **GTGAACGATCCAATACTATTGAGACGACTACAAAGGCTCAACTCGAAAAA**  **TCTCGCCGCGGAGCCAAGAAGAGGATAAAGGAGAGAGCATTGATGGGCTC**  **CCTTCACTCGACACTTAATGGAAATTCGATTGCCGGATCGATTCAAACGA**  **TTTCTCACGATTTGTATGATGATGATTCAATGCAAGGAGCATTTGATAAC**  **GTTCCATCATCTTTCCGTCCCCGAACTCAATCGAACCTCTCGATTCCTGG**  **ATCGTCGTCTCGTGTTTCTCCAGCTATTGGAAGTGATATCTATGATGATC**  **TAGAATTCCCATCATGGGTTGGNCGAATCCGGTTCCAGCAATTTCCAAGT**  **GANNTTGGTTGATAAGAACTGGATCAAATGNNNNNCNGATGNANCTACNC**  **NNTNTTGGTGGNNTTNNAGATTANNCCGGNAGTCCAANGCCCNNTTAANA**  **ACNNACCCNANTTGGNNNNNNNNGNNNNNNNNNCCNNNNGNTTGAAAANN**  **NNNNCCANGGNANCCGGGGGNTTCCNNANNCNNNTNNNNNNCNAANTNCC**  **NNTTGGTNCCNATNNNGNNNNNTNNNANNNNNNNNGNCNNNCNACCGGNN**  **NGCACNNGGNNNNNNCTNNTNNAANTGNGTGGCAAANNNNNNNNNNNNAN**  **TNNNN** |

**Table 3.** Total number of animals used for lifespan experiments**.**

| **Lifespan (Crude extract)** | **Total number of animals at the beginning of the experiment** | **Total Censored** | **Actual number of animals used** |
| --- | --- | --- | --- |
| Replicate 1 | 176 | 41 | 135 |
| Replicate 2 | 176 | 37 | 139 |
| Replicate 3 | 176 | 42 | 134 |
| **Lifespan (RNAi)** | **Total number of animals at the beginning of the experiment** | **Censored** | **Actual number of animals used** |
| Replicate 1 | 176 | 44 | 132 |
| Replicate 2 | 176 | 39 | 137 |
| Replicate 3 | 176 | 41 | 135 |
